# Comparison of heart-sparing radiation techniques for left-sided breast cancer: DIBH combined with tangential 3D radiation vs. conventional and tangential VMAT techniques

**DOI:** 10.1186/s13014-026-02807-y

**Published:** 2026-02-19

**Authors:** Kathrin Burchardt, Mirko Fischer, Michael Bremer, Roland Merten, Anne Caroline Knöchelmann

**Affiliations:** Department of Radiotherapy, Hannover Medical School, Hannover, Germany

**Keywords:** Breast cancer, Hypofractionated radiotherapy, Deep inspiration breath hold, Tangential VMAT

## Abstract

**Background:**

Adjuvant radiotherapy after breast-conserving surgery reduces local recurrence and improves survival in breast cancer. For left-sided tumors, achieving homogeneous target coverage while minimizing dose to organs at risk (OARs), particularly the heart and ipsilateral lung, remains challenging. The German S3-Guideline recommends hypofractionated whole-breast irradiation combined with deep inspiration breath hold (DIBH) to limit heart dose. This planning study compared conventional 3D conformal tangential radiotherapy (3D-FiF) and continuous-arc VMAT (cVMAT) with a novel tangential partial-arc VMAT technique (tVMAT) in left-sided breast cancer.

**Methods:**

Twenty patients with left-sided breast cancer were included. For each patient, three DIBH treatment plans were generated: 3D-FiF with two opposed tangential fields and two subfields, cVMAT with a 240° double arc, and tVMAT with two partial double arcs of 40° each. Dose differences were evaluated using the Wilcoxon signed-rank test.

**Results:**

Dose coverage of the clinical target volume (CTV) (D_95%_) was comparable between 3D-FiF and tVMAT, while cVMAT required a higher dose. tVMAT achieved a more homogeneous CTV dose distribution and showed significantly better conformity for both planning target volume (PTV) and CTV compared to 3D-FiF. Compared to cVMAT, tVMAT provided similar conformity and homogeneity for the PTV but lower mean doses to the heart, LAD, and left ventricle. 3D-FiF resulted in the lowest low-dose exposure of the lungs and contralateral breast, while tVMAT reduced these doses by factor 2.06 for the contralateral breast and factor 2.38 for the contralateral lung.

**Conclusions:**

tVMAT proved to be a robust and reproducible irradiation technique for left-sided whole-breast irradiation in DIBH using a hypofractionated protocol. While achieving excellent dose homogeneity and conformity in the target volume, OARs – especially cardiac and contralateral structures – received significantly less low-dose exposure compared to cVMAT. Compared with 3D-FiF, tVMAT achieved more homogeneous and conformal dose distributions and was less dependent on individual planner experience. Our findings encourage the implementation of tVMAT to achieve optimal target coverage with minimal organ-at-risk exposure.

**Clinical trial registration:**

Not applicable.

## Background

With approximately 70,550 new cases annually, breast cancer accounts for about 30% of all cancers in women in Germany [1]. Once the diagnosis is confirmed, about 70% of patients are eligible for breast-conserving surgery (BCS) [2]. Following surgery, adjuvant radiotherapy is an important tool to lower the recurrence risk and increase the overall survival (OS) [3, 4]. Irradiation of the left breast poses significant challenges for radiation oncologists who aim to ensure uniform coverage of the concave target volume while keeping the dose to nearby organs at risk (OARs) as low as reasonably achievable, especially to the lungs, the heart and the contralateral breast. To lower the dose to the heart especially in women with left sided breast cancer, the current German S3-Guideline [5] recommends irradiation with the aid of breath-holding techniques (deep inspiration breath hold, DIBH). Delivering irradiation to the left breast with DIBH is proved to be an accessible and effective method to reduce the radiation dose to the heart [6]. In addition, the low dose exposure of any healthy tissue should be kept as low as possible to lower the risk of stochastic radiation damage such as secondary malignancies [7].

Conventional 3D tangential irradiation (3D-CRT) offers good dose sparing in the contralateral lung and contralateral breast, while the dose to the heart and the ipsilateral lung can be improved by rotation techniques. The volumetric modulated arc therapy (VMAT) technique lowers the dose received by the heart and the ipsilateral lung, but at the same time increases radiation dose to the contralateral breast and contralateral lung [8–11]. Radiation treatment planning is highly individualized and depends not only on the patient’s anatomy and on the radiation technique used, but also depends on the experience of the radiation oncologist and the involved medical physicist and the amount of time they can spend on creating individual plans [12, 13]. In clinical practice, it is desirable to have a treatment plan that is created as efficiently as possible and is as independent as possible of the experience of the treating physician.

An approach to combine the advantages of the 3D-CRT and the VMAT techniques with the aim to keep the radiation burden to the organs at risk as low as reasonably achievable is the irradiation in VMAT technique with two partial arcs, as previous described by Virén et al. [10]. They evaluated a tangential VMAT technique for left-sided breast cancer radiotherapy and found that it provided excellent target coverage with improved dose conformity. Compared to conventional tangential techniques, tangential VMAT significantly reduced radiation exposure to the heart and lung, suggesting it is a promising approach for minimizing treatment-related toxicity.

In this planning study we compared the established irradiation techniques 3D-CRT and VMAT with a continuous arc (cVMAT) with the novel approach of VMAT with two partial, tangential arcs (tVMAT) in patients with left sided breast cancer and irradiation in DIBH based on a hypofractionated protocol.

## Methods

### Patients

In this retrospective planning study, 20 female patients who had undergone BCS for left-sided breast cancer and were eligible for adjuvant radiotherapy using DIBH between October 2020 and September 2023 were included. Patients who had undergone mastectomy or had breast implants were excluded. The median age was 63.5 years (range 34–74 years). Patients were selected retrospectively and without predefined selection criteria to provide a representative sample of our institutional patient population.

### Delineation of target and OAR

The contour of the clinical target volume (CTV) included all glandular and fibroglandular tissue within the left breast excluding skin, chest wall muscles and ribs, analog the European Society for radiotherapy and oncology (ESTRO) guidelines [14]. Towards the skin CTV was cropped by 5 mm. The planning target volume (PTV) was created by adding a 5 mm margin to the CTV and excluded the left lung. Towards the skin PTV was cropped by 3 mm, as customary in our clinic to avoid radiation damage to the skin.

The right breast was also contoured analog the ESTRO guidelines, including all breast tissue [14]. The left and the right lung where contoured according to anatomy. The heart, the left atrium, the left ventricle and the left anterior descending artery (LAD) where contoured analog Feng et al. [15].

### Treatment

All plans were based on imaging acquired using a Siemens Somatom Definition AS computer tomography (CT) scanner. The scans displayed the thorax, including the entire lung, at a secondary reconstruction interval of 3 mm. The reconstructions were made using the soft kernel. Patients lay in supine position on an IT-V BreastSTEP system (Manufacturer: Innovative Technik Völp, Innsbruck, Austria) with the arms placed above the head. The CT scans were performed in DIBH.

**Fig. 1:**
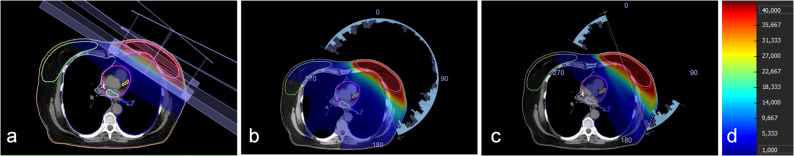
Beam arrangement and representative dose coverage for the three used radiation techniques in one sample patient. **a**) 3D tangential irradiation field-in-field **b**) continuous VMAT **c**) tangential VMAT **d**) color code from 1–40 Gy

The treatment plans were created for Elekta Versa HD linear accelerator (Elekta AB, Stockholm, Sweden) equipped with an Agility multileaf collimator with 160 leaves and 5 mm leaf width at isocenter. For each patient we created 3 treatment plans (Fig. 1): (1) 3D-CRT with two opposed tangential fields and two subfields (field-in-field technique, 3D-FiF) for dose modulation. The angle of the gantry and the collimator was individually placed for each patient. (2) Continuous VMAT (cVMAT) with a 240° arc with a double rotation. All cVMAT plans used identical arc geometry with a gantry start at 110°. (3) 2 partial double arcs with 40° each that are individually positioned analog the 3D-FiF tangential fields (tVMAT). An energy of 6 MV photon beam was used in all planning techniques.

All treatment plans were generated using the Monaco treatment planning system (version 6.2.2., Elekta). The prescribed dose was 40.05 Gy in 15 fractions to the PTV (2.67 Gy per fraction). Treatment planning was conducted based on the criteria outlined in ICRU reports 50, 62 and 83 [16–18]. During planning the main focus was to achieve adequate target coverage, defined as at least 95% of the CTV receiving 95% of the prescribed dose (38.05 Gy). In addition, less than 2% of the PTV should receive less than 107% of the prescribed dose (42.85 Gy) and uniform dose distribution in the PTV was aimed for. The dose received by OARs was supposed to be as low as reasonably achievable and whenever possible should stay within our institutional planning constraints (Table 1), which were adjusted according to Timmerman et al. [19].

**Table 1:** Institution-specific constraints in radiotherapy planning

Structure	Parameter	Constraint
PTV	D_2%_	≤ 42.85 Gy
CTV	V_40Gy_	≤ 400 cm³
	D_95%_	≥ 38 Gy
Breast, contralateral	D_2cm³_	≤ 6 Gy
	D_mean_	≤ 2 Gy
Lung, ipsilateral	V_20Gy_	≤ 15%
	V_15Gy_	≤ 20%
Lung, contralateral	V_3Gy_	≤ 8%
Heart	D_mean_	≤ 2.5 Gy
LAD	D_0.01 cm³_	≤ 20 Gy
	D_mean_	≤ 7 Gy
Left atrium	D_mean_	≤ 3 Gy
Left ventricle	D_mean_	≤ 3 Gy

PTV: planning target volume. CTV: clinical target volume. LAD: left anterior descending artery

Each radiation plan was evaluated and compared according to the following criteria: target coverage (D_2%_ of the PTV, D_95%_ of the CTV), the homogeneity index (HI), the conformity index (CI) and the radiation dose received by the OARs. Dose-volume histograms (DVHs) were used for visual and quantitative analysis. In literature several ways to determine the HI can be found, but following formula was calculated in this study to compare homogeneity:

$$\:HI=\frac{{D}_{2\%}}{{D}_{95\%}}$$

where D_2%_ is the minimum dose received by 2% of the PTV and D_95%_ is the minimum dose received by 95% of the PTV. The closer the value of the HI is to 1, the more homogeneous the dose is distributed within the target volume [19].

We used the CI as defined by Paddick [20], which is

$$\:CI=\frac{\left(T{V}_{PIV}\right)^{2}}{TV*PIV}$$

where TV_PIV_ is the target volume covered by the prescription isodose, TV is the target volume and PIV is the total volume covered by the prescription isodose. The closer the value of the CI is to 1, the higher the conformity of the plan is, meaning the better the plan covers the target volume without any excess of the prescribed dose or underdosage of the target volume [21].

We also compared all three planning techniques by monitor units (MUs) to assess delivery efficiency, with lower MUs indicating shorter treatment times and reduced scatter radiation potentially minimizing unintended dose to surrounding healthy tissue [7].

### Statistical analysis

Statistical analysis was performed using Jamovi (version 2.6.44). The non-parametric Wilcoxon signed-rank test was used to evaluate differences between the tVMAT and 3D-FiF plans, as well as between the tVMAT and cVMAT plans. A p-value < 0.05 was considered statistically significant.

Based on the 20 individual datasets, an average DVH was generated and subsequently fitted using a sigmoidal dose–response (inhibitory form, Inhibitor vs. normalized response) model with dose on a linear X-axis in GraphPad Prism (version 10.1.2).

## Results

### Target volume

The mean volume of the PTV was 822.2 cm³ with a range 389.8 cm³ to 1947.3 cm³.

The dose coverage of the CTV, represented by the D_95%_, which in this study is 38.05 Gy, was equal in 3D-FIF (mean 38.45 Gy ± 0.47 Gy) and tVMAT (mean 38.43 Gy ± 0.35 Gy). In cVMAT, a significantly higher dose was needed than in the tVMAT to cover 95% of the CTV (mean 38.72 Gy ± 0.37 Gy, *p* < 0.05).

The tVMAT generated a more homogenious dose coverage of the CTV, as expressed in the HI, than both the 3D-FiF and the cVMAT (*p* < 0.05). For the PTV the HI of all three techniques showed no statistical significance (p 0.131 for tVMAT vs. 3D and p 0.761 for tVMAT vs. cVMAT). The tVMAT plans showed significantly better CI in both the PTV and the CTV (*p* < 0.05) than the 3D-FiF plans, while in comparison with the cVMAT plans they showed no statistical difference for the PTV (*p* < 0.05). The CI of the CTV was significantly improved in the cVMAT plans compared to the tVMAT plans (*p* < 0.05) (Tables 2 and 3).

**Table 2:** Dosimetric comparison of the means with standard deviation of the target volume and the surrounding organs at risk in 3D-FiF and tVMAT plans (*n* = 20)

Structure	Parameter	3D-FiF	tVMAT	*p*-value
PTV	D_2%_ [Gy]	42.01 ± 0.35	41.18 ± 0.15	< 0.001
	HI	1.13 ± 0.02	1.15 ± 0.03	0.131
	CI	0.79 ± 0.04	0.84 ± 0.05	0.004
CTV	D_95%_ [Gy]	38.54 ± 0.47	38.43 ± 0.35	0.546
	HI	1.09 ± 0.02	1.07 ± 0.01	0.002
	CI	0.69 ± 0.08	0.78 ± 0.08	< 0.001
Lung, ipsilateral	V_20_ [%]	6.92 ± 3.22	5.95 ± 3.00	0.012
	V_15_ [%]	8.23 ± 3.41	8.70 ± 3.45	0.202
	D_mean_ [Gy]	4.11 ± 1.14	4.50 ± 1.12	0.040
Lung, contralateral	V_3_ [%]	0 ± 0	0.34 ± 0.81	0.021
	D_mean_ [Gy]	0.34 ± 0.04	0.50 ± 0.11	< 0.001
Breast, contralateral	D_2cm³_ [Gy]	1.81 ± 0.61	3.04 ± 1.76	0.001
	D_mean_ [Gy]	0.59 ± 0.21	0.96 ± 0.49	< 0.001
Heart	D_mean_ [Gy]	1.11 ± 0.40	1.21 ± 0.34	0.050
LAD	D_0.1 cm³_ [Gy]	9.55 ± 11.61	5.18 ± 2.54	0.452
	D_mean_ [Gy]	3.67 ± 3.43	2.65 ± 0.81	0.985
Left atrium	D_mean_ [Gy]	0.62 ± 0.08	0.79 ± 0.09	< 0.001
Left ventricle	D_mean_ [Gy]	1.40 ± 0.78	1.41 ± 0.58	0.261
MUs	/ Fraction	309.95 ± 5.09	600.04 ± 62.21	< 0.001

PTV: Planning target volume. CTV: Clinical target volume. LAD: Left anterior descending artery. MUs: Monitor units. HI: Homogeneity Index. CI: Conformity Index

### Organs at risk

**Fig. 2:**
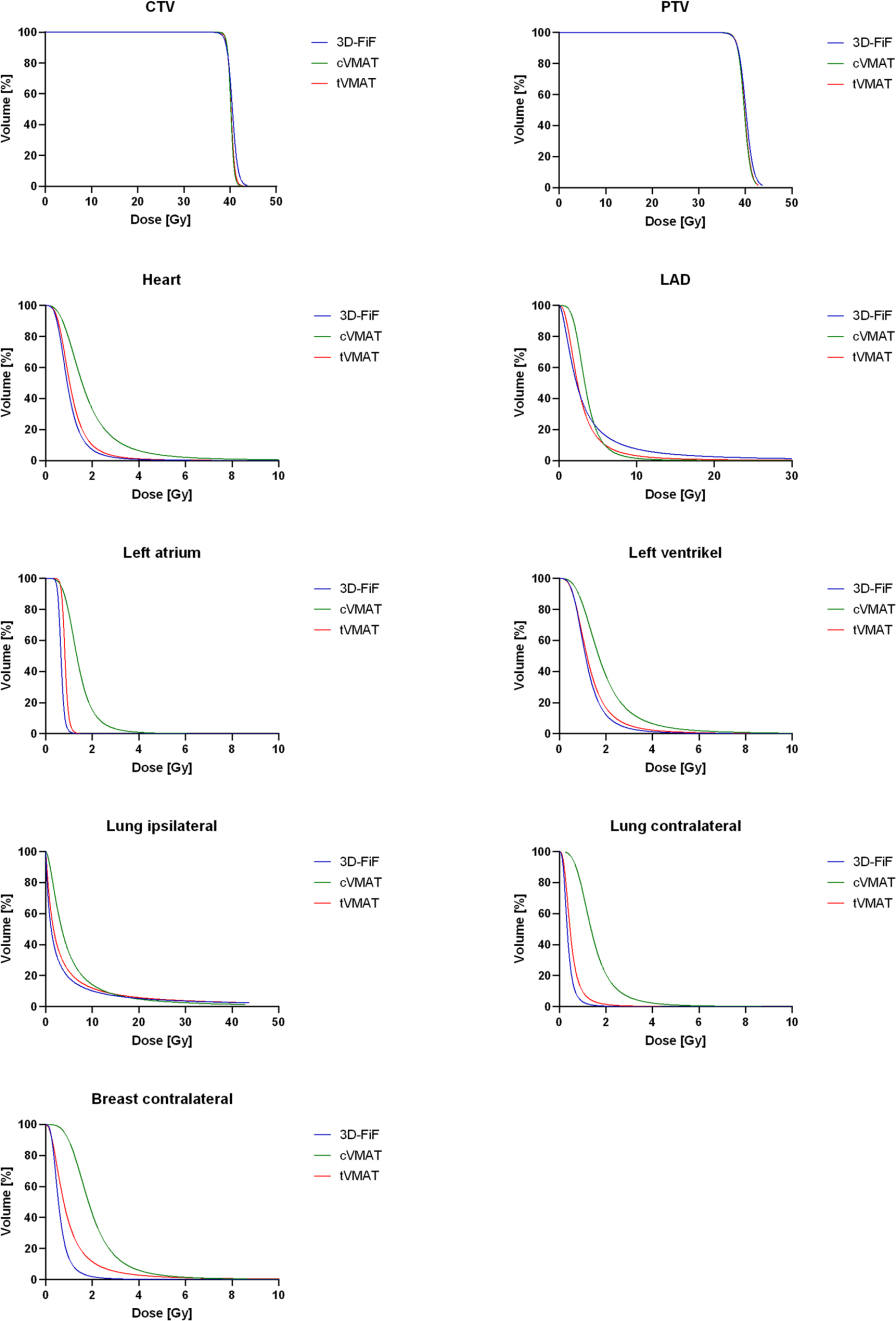
Cumulative dose–volume histograms of the three techniques averaged across all 20 patients. CTV: Clinical Target Volume. PTV: Planning Target Volume. LAD: Left anterior descending artery. 3D-FiF: 3D tangential irradiation field-in-field. cVMAT: continouos VMAT. tVMAT: tangential VMAT

There was no difference in the received mean dose of the heart, the LAD and the left ventricle in 3D-FiF and tVMAT (Table 2). The 3D-FiF plans showed lower low-dose radiation exposure of the lungs (ipsilateral lung D_mean_ 4.11 ± 1.14, contralateral lung D_mean_ 0.34 ± 0.04) as well as the contralateral breast (D_mean_ 0.59 ± 0.21) than both of the VMAT plans (cVMAT: ipsilateral lung D_mean_ 5.52 ± 1.31, contralateral lung D_mean_ 1.19 ± 0.14, contralateral breast 1.98 ± 0.33. tVMAT: ipsilateral lung D_mean_ 4.50 ± 1.12, contralateral lung D_mean_ 0.50 ± 0.11, contralateral breast 0.96 ± 0.49). Comparing the VMAT plans, the tVMAT lowered the mean dose of the contralateral lung by factor 2.38 and the mean dose of the contralateral breast by factor 2.06. The tVMAT also significantly lowered the mean dose of the heart with its substructures LAD, left atrium, and left ventricle compared to cVMAT. The only difference in a low-dose radiation exposure of cardiac structures between the 3D-FiF and the tVMAT was a lower mean dose to the left atrium in the 3D-FiF plans (0.62 Gy ± 0.08 Gy vs. 0.79 Gy ± 0.09 Gy, *p* < 0.001) (Tables 2 and 3; Fig. 2).

**Table 3:** Dosimetric comparison of the means with standard deviation of the target volume and the surrounding organs at risk in cVMAT and tVMAT plans (*n* = 20)

Structures	Parameter	cVMAT	tVMAT	*p*-value
PTV	D_2%_ [Gy]	41.10 ± 0.14	41.18 ± 0.15	0.036
	HI	1.15 ± 0.04	1.15 ± 0.03	0.761
	CI	0.83 ± 0.06	0.84 ± 0.05	0.983
CTV	D_95%_ [Gy]	38.72 ± 0.37	38.43 ± 0.35	0.003
	HI	1.06 ± 0.01	1.07 ± 0.01	0.003
	CI	0.81 ± 0.08	0.78 ± 0.08	< 0.001
Lung, ipsilateral	V_20_ [%]	5.03 ± 2.82	5.95 ± 3.00	0.004
	V_15_ [%]	8.03 ± 3.43	8.70 ± 3.45	0.422
	D_mean_ [Gy]	5.52 ± 1.31	4.50 ± 1.12	< 0.001
Lung, contralateral	V_3_ [%]	3.65 ± 1.70	0.34 ± 0.81	< 0.001
	D_mean_ [Gy]	1.19 ± 0.14	0.5 ± 0.11	< 0.001
Breast, contralateral	D_2cm³_ [Gy]	4.87 ± 0.92	3.04 ± 1.76	0.002
	D_mean_ [Gy]	1.98 ± 0.33	0.96 ± 0.49	< 0.001
Heart	D_mean_ [Gy]	1.91 ± 0.29	1.21 ± 0.34	< 0.001
LAD	D_0.1 cm³_ [Gy]	5.82 ± 1.99	5.18 ± 2.54	< 0.05
	D_mean_ [Gy]	3.40 ± 0.54	2.65 ± 0.81	< 0.001
Left atrium	D_mean_ [Gy]	1.40 ± 0.45	0.79 ± 0.09	< 0.001
Left ventricle	D_mean_ [Gy]	1.90 ± 0.05	1.41 ± 0.58	< 0.001
MUs	/ Fraction	656.80 ± 62.17	600.03 ± 62.21	< 0.001

PTV: Planning target volume. CTV: Clinical target volume. LAD: Left anterior descending artery. MUs: Monitor units. HI: Homogeneity Index. CI: Conformity Index

### Monitor units

The MUs were markedly lower in the 3D-FiF plans compared to both VMAT techniques (3D-FiF vs. tVMAT; *p* < 0.001). With 600.03 ± 62.21 MUs per fraction, the tVMAT plans demonstrated significantly fewer MUs than the cVMAT plans with 656.80 ± 62.17 MUs per fraction (*p* < 0.001).

## Discussion

Early diagnosis and improved systemic therapy give many patients a long-term prognosis. So modern radiotherapy for breast cancer has to minimize long-term toxicity while maintaining effective tumor control. In this context, our study highlights the advantages of tVMAT as a technique that obtains this balance effectively. Compared to 3D-FiF, tVMAT achieved significantly more homogeneous and conformal dose distributions within the CTV, with improved CI for the PTV, without compromising the dose to critical OARs. Importantly, no significant difference in mean dose to the heart, LAD or left ventricle was observed between tVMAT and 3D-FiF, supporting earlier findings from Virén et al. [10], who did not investigate irradiation in DIBH.

Compared with cVMAT, tVMAT demonstrated clear advantages, delivering substantially lower doses to multiple thoracic OARs, including the contralateral breast, contralateral lung, heart, LAD, left atrium and left ventricle, consistent with findings by Zhao et al. [22]. Specifically, we observed a reduction in mean dose to the contralateral breast and contralateral lung by factors of 2.06 and 2.38, while maintaining equivalent target coverage. This is a significant benefit, particularly for younger patients. These results align with the concept of using restricted arc geometry in tVMAT to avoid excessive irradiation of non-target tissues, an approach also supported by Munshi et al. [23].

Our findings support the integration of DIBH with tVMAT as a promising strategy for further dose reduction of the OARs. As shown by Yu et al. [24], combining tVMAT with DIBH leads to significant additional reductions in mean heart and lung doses. This is especially beneficial in cases where maximal OAR protection is critical or in patients for whom conventional techniques would exceed dose constraints.

In contrast to the work of other study groups [10, 22, 24–26], who employed normofractionated regimes, our study, like Nithya et al. [27] and Tang et al. [28], utilized a hypofractionated protocol, which has become the standard in adjuvant breast radiotherapy after BCS [5]. Unlike Nithya et al. [27], however, who did not incorporate DIBH, our study integrated DIBH in combination with tVMAT, with the idea of further reducing doses to cardiac and contralateral structures. Our results align with published data by Tang et al. [28], who also showed a reduction of dosimetric parameters of the OARs by combining tVMAT and DIBH. To our knowledge, ours is the first study comparing tVMAT with 3D-FiF and cVMAT in DIBH in left-sided breast cancer after BCS, and the first to investigate the doses received by cardiac substructures other than the LAD.

The importance of keeping the low-dose exposure to the heart and lungs as low as reasonably achievable is well established. Even modest doses to the heart have been shown to increase the long-term risk of ischemic heart disease, especially when the LAD and left ventricle are involved. These risks are particularly significant in younger breast cancer patients, who may be cured of their disease and live for several decades post-treatment, so they are more likely to experience late radiation-induced toxicity [29–33]. As Darby et al. [29] has shown, cardiotoxic effects of radiotherapy may only manifest years or even decades after treatment, making heart sparing a priority even when acute toxicity is absent. Similarly, increased lung doses are associated with the development of radiation pneumonitis [34, 35], a rare complication that remains a potentially serious side effect when ipsilateral lung doses are not tightly controlled.

As expected, the 3D-FiF technique demonstrated by far the lowest amount of MUs, as treatment portals are not segmented. Comparing the VMAT techniques, tVMAT utilized significantly lower MUs than cVMAT, which is accompanied by decreased scattered radiation and shorter treatment times. From a technical perspective, reduced MUs might lower machine wear and maintenance demands.

The dosimetric advantages our study found led to a change in clinical practice at our clinic. tVMAT with and without DIBH has been adopted as a regularly used technique for left-sided breast cancer in patients with higher concern for OAR exposure—such as younger women, those with cardiac comorbidities, HER2-targeted therapy or other cardiotoxic systemtherapy, or in scenarios where DIBH is not feasible because of comorbidities. Furthermore, tVMAT proved to be more robust and less dependent on individual planner skill compared to 3D-FIF. This is particularly interesting in clinical settings with limited staff or variable experience among physicians and medical physicist. While 3D-FiF relies heavily on manual beam arrangement and planner expertise, tVMAT allows for a more standardized and automated planning approach, reducing inter-observer variability. In favour of 3D-FiF technology, it should be mentioned that it is more robust against the interplay effect due to its open portals, although its clinical relevance is very low [36].

Several limitations to our work should be acknowledged. First, our study is primarily dosimetric and does not include long-term clinical outcome data such as rates of local control, cardiac or pulmonary toxicity, or patient-reported quality of life. Prospective clinical trials with extended follow-up are necessary to validate the clinical benefits suggested by the dosimetric improvements we have seen. Second, while we used hypofractionation with DIBH, comparisons to other fractionation schemes or breathing techniques remain limited, as most previous studies have investigated normofractionated regimens without DIBH. Since even the plans of different radiation techniques within one study are challenging to compare and in need of a clear objective ahead of planning, it needs a lot of attentive thought to compare plans across independent studies with different set-ups. Third, as also discussed by Karpf et al. [24], it is still unclear whether improved homogeneity and conformity (HI and CI) translate into better clinical outcomes, such as lower recurrence rates or reduced toxicity. To date, no conclusive evidence links these dosimetric parameters directly to clinical endpoints, and further prospective studies are required to validate their predictive value. Fourth, patient-specific anatomical variations might still necessitate individualized planning approaches, and the optimal selection criteria for tVMAT versus cVMAT require further investigation. Finally, as patients were retrospectively selected without predefined criteria, a potential selection bias cannot be fully excluded. Nevertheless, this approach was chosen to reflect a representative real-world patient population treated at our institution.

## Conclusion

In summary, our data reinforce the role of tVMAT as a technically robust, reproducible, and OAR-sparing alternative to conventional techniques in the adjuvant treatment of left-sided breast cancer. The significant reduction in low-dose exposure to contralateral and cardiac structures, combined with superior target conformity and planning efficiency, supports the integration of tVMAT—ideally in combination with DIBH—into clinical routine.

## Data Availability

The datasets used and/or analysed during the current study are available from the corresponding author on reasonable request.

## Abbreviations

3D: Three-dimensional
3D-CRT: Conventional 3D tangential irradiation
3D-FiF: 3D field-in-field technique
BCS: Breast-conserving surgery
CI: Conformity index
CTV: Clinical target volume
cVMAT: Continuous volumetric modulated arc therapy
CT: Computed tomography
D_0.01cm³_: Maximum dose received by the hottest 0.01 cm³ of a structure
D_2%_: Minimum dose received by 2% of the planning target volume
D_2cm³_: Dose received by the hottest 2 cm³ of a structure
D_95%_: Minimum dose received by 95% of the clinical target volume
D_mean_: Mean dose received by a structure
DIBH: Deep inspiration breath-hold
DVH: Dose-volume histogram
ESTRO: European Society for Radiotherapy and Oncology
Gy: Gray
HI: Homogeneity index
ICRU: International Commission on Radiation Units and Measurements
LAD: Left anterior descending artery
MUs: Monitor units
OAR: Organs at risk
OS: Overall survival
PIV: Total volume covered by the prescription isodose
PTV: Planning target volume
tVMAT: Tangential volumetric modulated arc therapy
TV: Target volume
TVPIV: Target volume covered by the prescription isodose
V_15Gy_: Volume of a structure receiving at least 15 Gy
V_3Gy_: Volume of a structure receiving at least 3 Gy
V_40Gy_: Volume of a structure receiving at least 40 Gy
VMAT: Volumetric modulated arc therapy
